# Collagenated Porcine Heterologous Bone Grafts: Histomorphometric Evaluation of Bone Formation Using Different Physical Forms in a Rabbit Cancellous Bone Model

**DOI:** 10.3390/molecules26051339

**Published:** 2021-03-02

**Authors:** Rui I. Falacho, Paulo J. Palma, Joana A. Marques, Maria H. Figueiredo, Francisco Caramelo, Isabel Dias, Carlos Viegas, Fernando Guerra

**Affiliations:** 1 Institute of Oral Implantology and Prosthodontics, Faculty of Medicine, University of Coimbra, 3000-075 Coimbra, Portugal; rifalacho@fmed.uc.pt (R.I.F.); fguerra@ci.uc.pt (F.G.); 2 Institute of Endodontics, Faculty of Medicine, University of Coimbra, 3000-075 Coimbra, Portugal; joanaamarques@uc.pt; 3 Center for Innovation and Research in Oral Sciences (CIROS), Faculty of Medicine, University of Coimbra, 3000-075 Coimbra, Portugal; 4 Dentistry Department, Faculty of Medicine, University of Coimbra, 3000-075 Coimbra, Portugal; mhfigueiredo@fmed.uc.pt; 5 Laboratory of Biostatistics and Medical Informatics (LBIM), Coimbra Institute for Clinical and Biomedical Research (iCBR), Faculty of Medicine, University of Coimbra, 3000-548 Coimbra, Portugal; fcaramelo@fmed.uc.pt; 6 Department of Veterinary Sciences, School of Agricultural and Veterinary Sciences, University of Trás-os-Montes e Alto Douro (UTAD), 5000-801 Vila Real, Portugal; idias@utad.pt (I.D.); cviegas@utad.pt (C.V.); 7 Centre for the Research and Technology of Agro-Environmental and Biological Sciences, CITAB-UTAD, 5000-801 Vila Real, Portugal; 8 ICVS/3B’s–PT Government Associate Laboratory, Braga/Guimarães, Portugal 3B’s Research Group, I3Bs–Research Institute on Biomaterials, Biodegradables and Biomimetics, Headquarters of the European Institute of Excellence on Tissue Engineering and Regenerative Medicine, University of Minho, 4805-017 Guimarães, Portugal; 9 Laboratory of Hard Tissues, Dentistry Department, Faculty of Medicine, University of Coimbra, 3000-075 Coimbra, Portugal

**Keywords:** bone grafting, bone regeneration, collagenated bone, histomorphometry, porcine bone graft, rabbit model

## Abstract

Collagenated porcine-derived bone graft materials exhibit osteoconductive properties and the development of different formulations intends to enhance bone regeneration. This study aims to evaluate bone healing in a rabbit cancellous bone defect in response to grafting with different physicochemical forms of heterologous porcine bone. Twenty-six adult male New Zealand White rabbits received two critical size femoral bone defects per animal (n = 52), each randomly assigned to one of the five tested materials (Apatos, Gen-Os, mp3, Putty, and Gel 40). Animals were sacrificed at 15- and 30-days post-surgery. Qualitative and quantitative (new bone, particle and connective tissue percentages) histological analyses were performed. Histomorphometry showed statistically significant differences in all evaluated parameters between mp3 and both Putty and Gel 40 groups, regardless of the timepoint (*p* < 0.05). Moreover, statistical differences were observed between Apatos and both Putty (*p* = 0.014) and Gel 40 (*p* = 0.007) groups, at 30 days, in regard to particle percentage. Within each group, regarding new bone formation, mp3 showed significant differences (*p* = 0.028) between 15 (40.93 ± 3.49%) and 30 (52.49 ± 11.04%) days. Additionally, intragroup analysis concerning the percentage of particles revealed a significant reduction in particle occupied area from 15 to 30 days in mp3 and Gen-Os groups (*p* = 0.009). All mp3, Gen-Os and Apatos exhibited promising results in terms of new bone formation, thus presenting suitable alternatives to be used in bone regeneration.

## 1. Introduction 

Nowadays one of the most pressing subjects in modern oral rehabilitation is the recovery of lost or resorbed bone architecture, aiming at a functional and aesthetic recovery [1,2]. Several surgical methods and graft materials or bone substitutes are available for bone defect regeneration. According to recent literature, no technique stands out in terms of clinical efficacy and the decision should vary according to case-specific diagnosis [3,4,5]. Several bone grafting techniques have been proven safe, present solid research data and are accessible to the clinicians [6,7,8,9,10,11,12].

Bone substitutes consist of any biomaterial, biologic or synthetic, intended for implantation in humans with the prospect of rebuilding bone mass, strengthening bone structure or filling bone loss [12,13]. Bone replacement materials that are available come essentially from four distinct origins: the individual himself (autogenous grafts), a different donor belonging to the same species (allogeneic grafts), donors belonging to another species (xenogeneic grafts), or synthetically produced materials (alloplastics). All bone graft biomaterial groups have disadvantages either related to the host reaction (immune responses), the quantity available, properties after manufacturing processes, rapid resorption, among others [14,15].

Currently, material selection for a given intervention is based on several factors such as the characteristics of the material itself, the type of bone defect to be treated, the operator’s preferences, the associated costs, and the patient’s acceptance. The range of clinical situations is wide, and a single material may not be the universal solution, but it is rather imperative to observe its characteristics, as well as its formulation and presentation, that may be best suited for each specific clinical condition. Although autologous bone is still considered the gold standard when it comes to bone substitutes [16,17], clinical success is not guaranteed and complications [18] may occur in 8–39% of cases [19]. Some main disadvantages of this type of graft are the unpredictability with regard to its resorption, the need for a second surgical procedure at the donor site and the amount harvested that may not be sufficient for some defects [20,21,22]. Allografts exhibit osteoinductive and osteoconductive activity, but lack osteogenic properties, since no viable cells are part of these bone grafts [18,23,24]. Xenogeneic bone grafts are materials from a different species and present an alternative to both autogenous and allogeneic grafts. These materials classically exhibit osteoconduction characteristics, being considered neither osteoinductive nor osteogenic. Some papers discuss whether this classical view might still be applied to new xenografts, or if these may have osteoinductive properties [25,26]. Most xenografts currently used have porcine and bovine origins due to their similarity to human bone regarding chemical composition and structure [23]. Porcine-derived xenografts underwent and still undergo a great deal of research to assess their potential as bone substitutes, as they originate in an animal species with a genotype close to human. Various studies have shown that such materials provide an effective osteoconductive matrix [26,27]. Nannmark et al. [28] have confirmed the good biocompatibility and osteoconductive properties of porcine bone. 

Undecalcified bone tissue samples and histomorphometric techniques revolutionized the understanding of bone structure and physiology [29,30]. In fact, histomorphometry is one of the crucial instruments for assessing bone tissue and its changes, as well as to evaluate mechanisms and repercussions of test materials on bone tissue, rendering helpful data about structure, formation, resorption, mineralization, as well as modeling and remodeling activity [30,31,32]. Histomorphometry is well suited for preclinical animal models that can provide substantial histological information through appropriate experimental methodologies. Animal models are favored when studying new materials or material presentations, as is the case of the experimental study portrayed in this paper which tests new formulations of a porcine derived bone substitute [33]. Regarding animal model selection, rabbit is one of the most frequently used animal models for medical research, comprising approximately 35% of musculoskeletal system research studies [34,35]. Rabbit’s simple handling and similar bone metabolism to humans, make it the first choice in evaluating bone graft materials [35,36].

Currently, the material selection for a given intervention is based on several factors such as characteristics of the material itself, type of bone defect to be corrected, operator’s preferences, ease of handling, associated costs and patient’s acceptance. The present study focuses on novel formulations of porcine-derived xenografts, with some offering new presentations that may render an easier handling and application with the possibility of maximizing technical performance if histological outcomes reveal promising.

The main purpose of the current study is to evaluate bone healing in a rabbit cancellous bone critical size defect in the lateral aspect of the distal femur in response to filling with five different physicochemical forms of heterologous porcine bone (Apatos, Gen-Os, mp3, Putty, and Gel 40).

The null hypothesis of this experimental work states that the five different porcine-derived bone graft materials exhibit similar histological and histomorphometric results.

## 2. Materials and Methods

### 2.1. Animal Study—Ethical Statement

This experimental protocol was approved by the national regulatory authority in animal research, as well as by the University of Trás-os-Montes and Alto Douro Ethics Commission (N° CE 29/2015). Animal housing and manipulation, as well as experimental procedures and data reporting followed Portuguese legislation related to the use of animals for experimental purposes (Decreto-lei n° 113/2013, de 7 de Agosto) and European Legislation Directives on the protection of animals used for scientific purposes (Directive 2010/63/EU of the European Parliament and of the Council of 22 September 2010).

### 2.2. Study Design—Sample Size

Sample size calculation was performed using G*Power software considering bone defect as the unit, sustained on previous study results obtained in the scientific work of Palma et al. [37]. In this study it was found that the smallest effect size (d = 1.24) refers to an average difference of 1.20 ± 3.35% of new bone percentage at 2 weeks. The largest effect size (d = 2.45) refers to an average difference of 14.40 ± 3.80% at 4 weeks.

Three possible levels of significance were considered, α = 0.01, α = 0.05 or α = 0.10. Three different power levels were also considered, 0.80 (1−β = 0.80), 0.90 and 0.95. A bilateral Student’s t-test of independent samples and an allocation ratio between groups of 1:1 was also used to calculate the sample size.

Based on the obtained data, a sample consisting of 26 adult male New Zealand White rabbits (*Oryctolagus cuniculus*), weighing around 5.2 ± 0.56 kg, were included. This sample was divided into two series—15 and 30 days—of 13 animals each. In each animal, two test sites were surgically created in the lateral aspect of the distal femur of opposite limbs, thus a total of 52 test sites constitute the sample for research.

### 2.3. Housing, Maintenance, Handling, and Animal Welfare

Animal selection, handling and maintenance were carried out at the University of Trás-os-Montes and Alto Douro Vivarium. Animal’s general health conditions were evaluated by qualified technicians and Veterinary Doctors of the same University. Each animal was registered on the day of arrival and tagged with a unique microchip number. Clinical evaluation of their condition and weight was made and animals were placed in individual cages, completing a quarantine period of no less than 1 week prior to any intervention. The animals were housed in standard individual cages with all conditions and ventilation appropriate to the particular species traits. Throughout the experimental study, animals were permanently monitored under standardized temperature, humidity, air renewal, and light (12 h of light and 12 h of dark) conditions.

Surgical procedures were performed at the Veterinary Hospital of the University of Trás-os-Montes and Alto Douro and before undergoing surgery, animals were moved to specific presurgical rooms to maintain their stress levels low, as well as those from all housed animals. After surgical procedures, animals were transferred to specific recovery cages until full awareness and locomotion capabilities were demonstrated and then moved to their respective standard cages for unrestricted locomotion. Commercially dry food with controlled contaminant concentrations, according to manufacturer, was provided once a day and water was supplied ad libitum. 

### 2.4. Experimental Groups

Tested materials were randomly distributed using a computer algorithm in relation to animal, anatomical location and placement sequence, thus keeping the animal number to a minimum necessary by decreasing the effects of individual variation.

Test materials were assigned to the following groups (Table 1):

- Apatos group—100% porcine cortical bone granules (600–1000 µm particles) without preservation of collagen (Apatos, Osteobiol^®^, Tecnoss, Torino, Italy)
- Gen-Os group—100% porcine cortico-cancellous bone granulated mix (250–1000 µm particles) with preserved collagen (Gen-Os, Osteobiol^®^, Tecnoss, Torino, Italy)
- mp3 group—90% porcine cortico-cancellous bone granulated mix (600–1000µm prehydrated particles) with preserved collagen with 10% collagen gel (mp3, Osteobiol^®^, Tecnoss, Torino, Italy)
- Putty group—80% porcine cortico-cancellous bone granulated mix (<300 µm micronized particles) with preserved collagen and 20% collagen gel (Putty, Osteobiol^®^, Tecnoss, Torino, Italy)
- Gel 40 group—60% porcine cortico-cancellous bone granulated mix with preserved collagen (<300 µm micronized particles) and 40% type I and III collagen gel (Gel 40, Osteobiol^®^, Tecnoss, Torino, Italy)

### 2.5. Anesthesia

Animals went through a twelve-hour fasting period, maintaining only water supply, before anesthesia which was initially induced using medetomidine (0.15 mg/kg SC) and butorphanol (0.1 mg/kg IM). Fifteen minutes after this initial procedure, the marginal ear vein was located, a gentle local trichotomy was made, and a slow injection of ketamine (5 mg/kg IM or EV) was administered. Endotracheal intubation was then performed with a small endotracheal tube in order to maintain a controlled anesthetic state by continuous inhalation of isoflurane (0.25–2%) in an oxygen flow of 0.5–2 L/min/kg. Monitoring throughout the surgical procedure was done using a noninvasive oximeter (respiratory rate and *oxygen-saturated hemoglobin*), a capnograph and cardiac auscultation.

Surgical procedures were performed under standard hospital aseptic rules, in a surgical ward of the Veterinary Hospital—University of Trás-os-Montes and Alto Douro.

### 2.6. Surgical Protocol

The area of interest was trichotomized, the animal was placed on a heated operating table in lateral decubitus and the limb was immobilized and suspended without excessive pressure to expedite disinfection procedures and allow for an easier establishment and maintenance of the sterile surgical field. Both surgeons then started preparing the surgical table, disinfecting the surgical field with a povidone-iodine solution (Betadine^®^, Mylan, Lisbon, Portugal) and ensuring adequate sterilization conditions for the surgery. During surgical procedures, only the intervention zone was exposed.

Surgery was performed on both hind limbs. A cutaneous incision was made in the lateral face of the stifle joint, over the distal femur and dissection made by planes with the help of dissection scissors and instruments. The fascia lata was incised at the level of the stifle retinaculum, followed by the medial luxation of the patella with the vastus lateralis muscle retained cranially and the biceps femoral muscle retracted caudally, in order to expose the lateral condyle of the femur. Upon periosteum exposure, a scalpel was used to section this membrane and a periosteal elevator to expose bone surface.

Subsequently, a critical size cylindrical epiphysis bone defect (5 mm in diameter by 10 mm in depth) was created in each test site (n = 52), in the lateral aspect of the distal femur in both limbs, through the distolateral cortex of the stifle joint without compromising the medial cortical bone and joint biomechanics. The volume of material placed on each bone defect was approximately 0.2 cc (V = πr2h).

Only one negative control per group was used, as critical size bone defects are already sufficiently established and referenced in the literature, requiring no model validation and, thus, reducing the number of animals [38,39].

**Figure 1:**
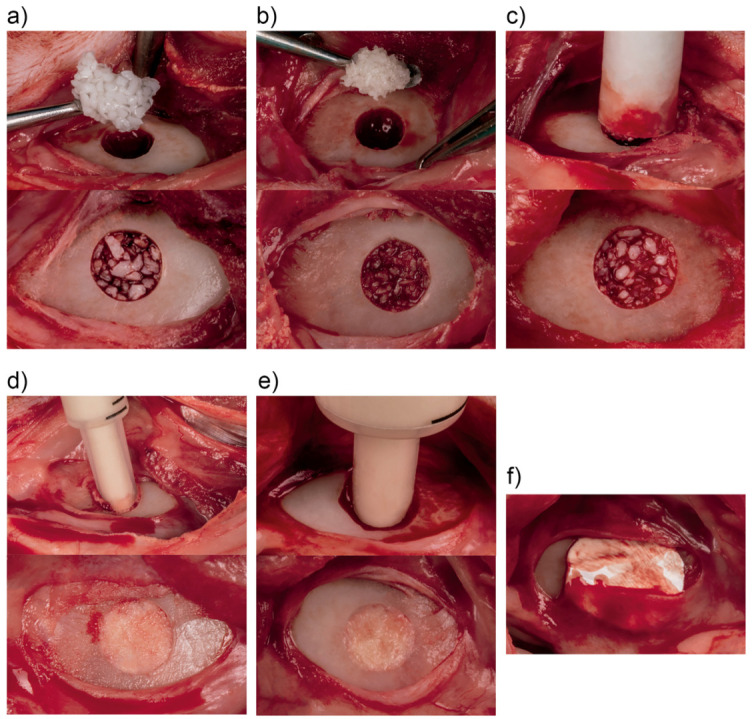
Intraoperative photography of material placement: (**a**) Apatos; (**b**) Gen-Os; (**c**) mp3; (**d**) Putty; (**e**) Gel 40; (**f**) Evolution membrane.

Meanwhile, Apatos group and Gen-Os group tested materials were hydrated with saline. All other test materials were prehydrated in vial; thus, no additional hydration procedure was required. After copious bone defect irrigation with saline to remove the debrided bone, experimental materials were placed gently but firmly at the defect site without over-condensing (Figure 1a–e), according to the randomization. In order to avoid or minimize material displacement and mimic clinical situations as closely as possible, a heterologous pericardium membrane with 100% preserved collagen (Evolution, Osteobiol^®^, Tecnoss, Torino, Italy) was shaped, hydrated and applied over the filled bone defect, overlapping its margins (Figure 1f). 

Soft tissue closure for each surgical site was performed by layers in order to maximize wound closure and regeneration, as well as to hold the biomaterial in place. Resorbable sutures were used on the periosteum and muscle layers, followed by the skin with nonresorbable stitches.

### 2.7. Postoperative Care

Immediately after surgery, a butorphanol tartrate dose (0.2 mg/kg, s.c., 2 days) was administered for pain control. A single long-term amoxicillin dose (1 mg/kg, s.c.) was also administered. 

Animals were placed in individual recovery cages at a dark, quiet, comfortable, clean location with controlled temperature and free of objects or materials that potentially could cause any harm when animals began attempts to move. When necessary, body temperature was maintained with the aid of thermally insulating wrapping materials. When full capacity for consciousness and locomotion was observed and anesthetic recovery was achieved, animals were then transferred to their standard cages with unrestricted movement and free access to food and water. Daily monitoring was conducted to evaluate any changes in food or water intake, body weight, typical ethological patterns of the species and for the presence of abnormal signs or adverse reactions, as well as pain.

### 2.8. Animal Euthanasia and Necropsy

At the end of each experimental period (15 and 30 days postoperatively), animals were euthanized individually, in an isolated room with no sensory access to other animals, by administering a lethal dose of ketamine hydrochloride. Animal necropsy was performed to assess the systemic impact of the biomaterials under study. After macroscopic analysis, fragments of the main organs (heart, liver, lungs, kidneys, spleen, and regional lymph nodes) were collected and processed for studies. The corpses and waste from euthanasia were subsequently incinerated in accordance with current legislation.

### 2.9. Study Material Harvesting

The material was harvested en bloc after meticulous soft tissue dissection. Collected samples were processed with an undecalcified technique, providing high-quality histological images, without morphological distortions of relevant structures or meaningful artefacts with the high-precision Exakt^®^ system (Exakt Technologies, Oklahoma City, OK, USA). Regarding staining methods, toluidine blue was used.

### 2.10. Sample Processing and Analysis

A qualitative and quantitative analysis of the processed study material was performed. For both analyses, slides obtained by the histological processing were observed using a stereomicroscope (Nikon^®^ SMZ 1500, Tokyo, Japan) and an optical microscope (Nikon^®^ Eclipse E600, Tokyo, Japan) in order to produce photographic records of the observations. Qualitative analysis focused mainly on the observation and recording of histomorphological tissue characteristics, bone/biomaterial interface, and the presence or absence of inflammatory response. The quantitative histological analysis, histomorphometry, was performed based on the image analysis software Bioquant^®^ Nova (Bioquant—Image Analysis Corporation, Nashville, TN, USA) which calculates the area of different tissues based on color variation. The same operator performed all calibration procedures, as suggested in literature [40].

### 2.11. Statistical Analysis 

Statistical analysis was performed using the commercially available IBM^®^ SPSS^®^ v24 software and MS^®^ EXCEL^®^. The significance level was set at α = 0.05. The description of the results was initially performed regarding the mean, standard deviation, maximum and minimum values of the measured parameters (new bone, particles and connective tissue), according to both biomaterial and period of evaluation. Additionally, descriptive statistics included the construction of dispersion diagrams for each evaluated parameter.

Kruskal–Wallis and Mann–Whitney nonparametric tests were used to detect significant differences in the median across the groups. Subsequently to Kruskal–Wallis testing, Dunn–Sidak post-hoc testing with correction for multiple comparisons was carried out. It should be noted that, although there were measures taken in two different times, the animals were different and, therefore, only independent tests were used.

This report follows the Animal Research: Reporting of In Vivo Experiments Guidelines (ARRIVE Guidelines) [39].

## 3. Results

### 3.1. Clinical Findings

The postoperative period was uneventful, with no systemic or local complications resulting from the surgical intervention being detected. Rapid recovery with healthy ethological behaviors revealed a reduced impact of the experimental protocol on animal health and welfare. Macroscopic observations of the en bloc collected femurs, showed no morphological changes or the existence of inflammatory processes beyond those expected and resulting from the surgical procedure. The histopathological analysis of collected organs showed no anatomopathological abnormalities.

### 3.2. Qualitative Histological Analysis

#### 3.2.1. Apatos Group 

**Figure 2:**
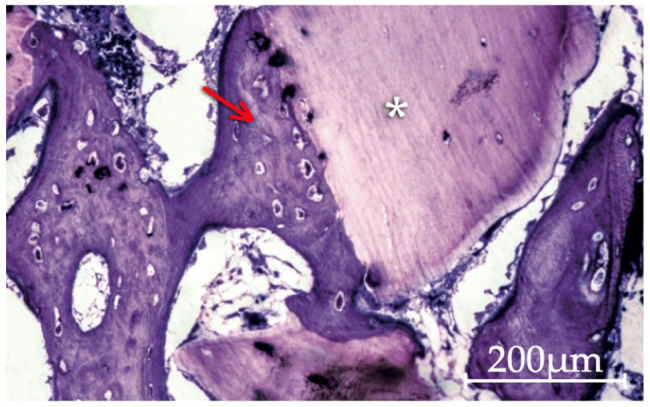
Apatos particles (asterisk) coated by a very immature bone tissue (arrow) formed by direct apposition to the biomaterial surface at 15 days. Areas of lax connective tissue occupying much of the interparticle spaces are still visible (200× original magnification).

The set of analyzed bone defect cross-sections showed a sequential and centripetal process in which trabeculae of new bone coated, integrated and bound the particles among themselves and to the defect walls. At 30 days, large areas of immature bone tissue can be found, along with some lamellar bone and intense bone formation activity, thus indicating an ongoing remodeling process (Figure 2).

#### 3.2.2. Gen-Os Group 

All analyzed bone defect cross-sections displayed a remarkable and intense osteoclastic activity, often adjacent to also significant amounts of osteoblast cells and osteoid matrix related to osteogenic phenomena.

**Figure 3:**
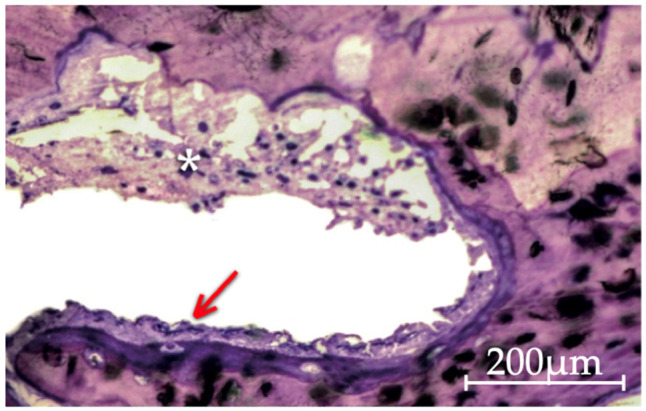
Histological image of the Gen-Os group at 30 days of evolution, showing a bone particle resorption process, with the creation of Howship’s lacunae by osteoclasts (asterisk), side by side with immature bone tissue formation processes by apposition to the particles, still with the presence of osteoblasts and osteoid (arrow) (200× original magnification).

Between 15 and 30 days, there is a clear difference in terms of bone remodeling phenomena shown by the notable areas of immature and some lamellar bone tissue trabeculae that colonize interparticle spaces but also replace the particles themselves (Figure 3).

#### 3.2.3. mp3 Group 

**Figure 4:**
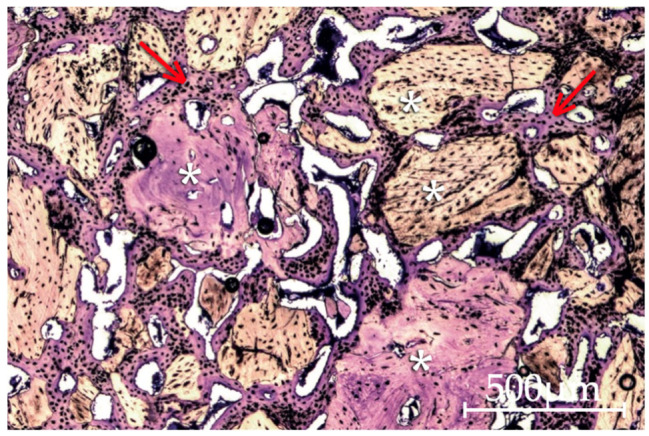
Image of the mp3 group at 30 days of evolution. The formation of an extensive network of bone trabeculae (arrow) is notorious, integrating and linking the bone graft material particles (asterisk). A certain heterogeneity in the shape, dimensions and tinting characteristics of the mp3 particles may also be perceived (40× original magnification).

**Figure 5:**
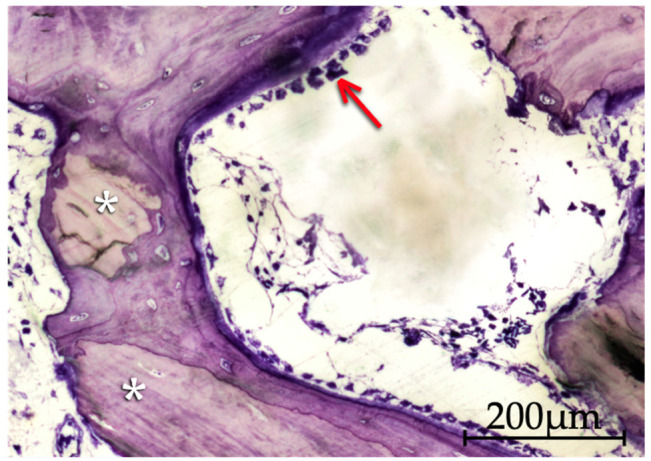
Histological aspect of mp3 particles at 15 days of evolution, surrounded by new apposition formed bone. The presence of a substantial number of osteoblasts indicates that synthesis activity is still ongoing. The image shows bone trabeculae containing several osteointegrated particles (asterisk) and a peripheral zone with prominent osteoblastic activity (arrow) (200× original magnification).

All bone defect transversal sections displayed intense bone neoformation with extensive bone trabeculae networks. At 15 days the central region of the defect already presented bone formation. At both timepoints, bridging phenomena are observed, with newly formed bone tissue integrating and connecting graft particles both on the peripheral and central areas of the defects. At 30 days lamellar bone tissue is more prevalent. Intense osteogenic activity is recorded at both timepoints with numerous active osteoblasts and osteoclasts. This activity is inferred by the presence of osteoblasts characterized as large mononucleated cells with an ovoid profile and abundant basophil cytoplasm, reflecting a high rate of protein and proteoglycan synthesis, often arranged in single cell lines along the immature bone surfaces, thus believed to synthesize organic components of bone matrix (osteoid) prior to their mineralization. The cell-mediated process of particle resorption was visible (Figure 4 and Figure 5).

#### 3.2.4. Putty and Gel 40 Groups 

The results shown for both groups in both timepoints have almost no variation in terms of tissue quality, with particle dispersion and migration being visible in both materials. Few to no bone formation is observed beyond the peripheral zones of the defect at both 15 and 30 days of evolution. 

### 3.3. Quantitative Histomorphometric Analysis

**Figure 6:**
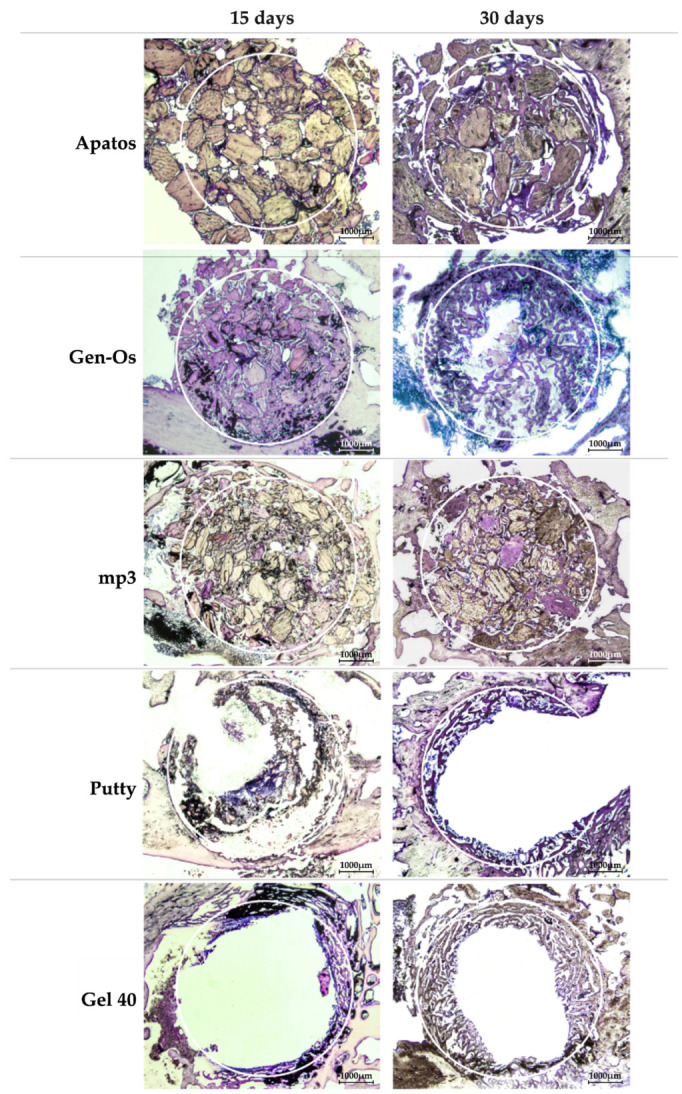
Cross section images of defects at 15 and 30 days of evolution, filled with the tested materials (15× original magnification).

Histomorphometric analysis was based on cross sections showing the entire defect, as shown in Figure 6.

#### 3.3.1. Analysis within Each Group

**Figure 7:**
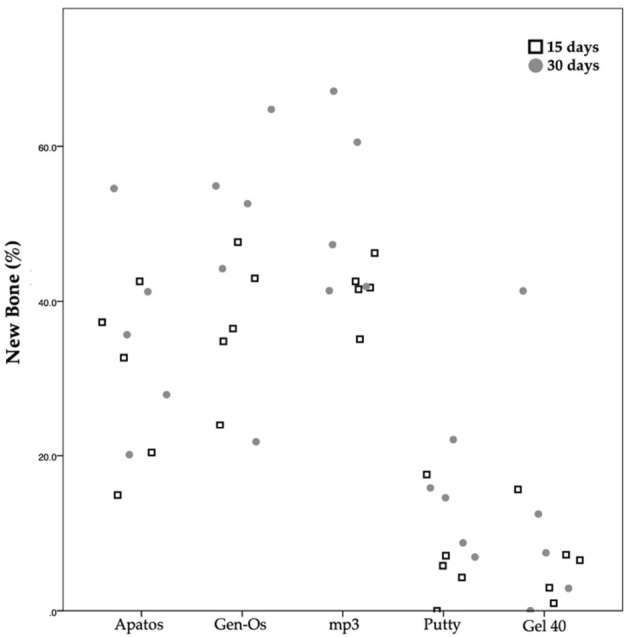
Scatter plot graph displaying the percentage of new bone measurements for different materials and at the two assessment times.

Regarding new bone formation (Figure 7), the comparison between 15 and 30 days within each group shows that mp3 is the only biomaterial in which a statistically significant difference was detected (*p* = 0.028), with a percentage of new bone at 30 days (52.49 ± 11.04%) being statistically higher than the percentage of new bone observed at 15 days (40.93 ± 3.49%). 

**Figure 8:**
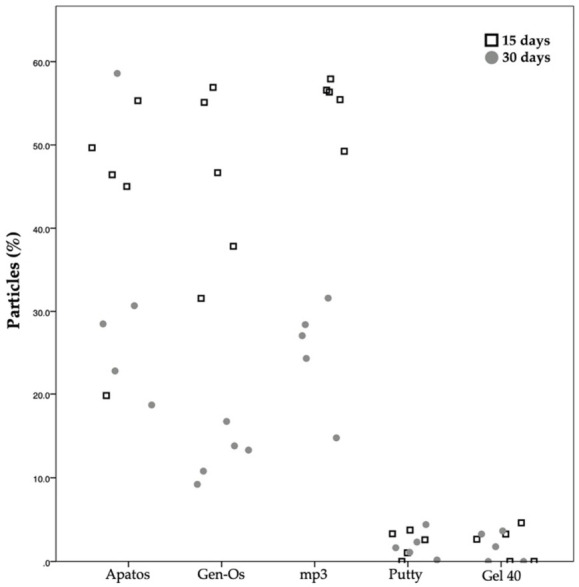
Scatter plot graph displaying the particles measurements for different materials and at the two assessment times.

Concerning the percentage of particles (Figure 8) in the defect at 15 and 30 days, within each group, statistically significant differences are observed in the mp3 group (*p* = 0.009) and Gen-Os group (*p* = 0.009), with a reduction in particle occupied area percentage from 15 to 30 days. 

#### 3.3.2. Analysis between Groups

At 15 days of evolution, comparing the groups among each other, the mp3 group presented statistically significant differences with the Putty and Gel 40 groups, independently of the analyzed parameter (*p* < 0.05).

At 30 days, considering the percentage of new bone (Table 2), statistically significant differences are detected between the mp3 group and both the Putty (*p* = 0.035) and Gel 40 (*p* = 0.015) groups. Regarding the percentage of particles (Table 3), statistically significant differences were observed between the mp3 group and both the Putty (*p* = 0.022) and Gel 40 (*p* = 0.011) groups, as well as between the Apatos group and the Putty (*p* = 0.014) and Gel 40 (*p* = 0.007) groups.

Taking into consideration these results, the null hypothesis stating that the five different porcine-derived bone graft materials exhibit similar histological and histomorphometric results, is then rejected.

## 4. Discussion

This experimental study was designed with the aim of understanding and evaluation of the bone regeneration potential of different bone graft materials in a physically contained bone defect. Formulations with isolated particles needing hydration and new formulations containing different carriers and particle concentrations were evaluated.

Although the cellular response to different biomaterials is based on in vitro studies, these tests do not accurately mimic in vivo realities. On the other hand, human models for bone regeneration studies have some major inconveniences such as the impracticality of performing adequate histological evaluations, even in cases where a biopsy can be performed, as well as challenges designing a study with satisfactory internal validity [1,33]. Therefore, preclinical animal models that can provide substantial histological information through appropriate experimental methodologies become an essential study method [33]. In order to respect the replacement, refinement or reduction (the 3Rs) principles for the use of animals in research, the present experimental study performed preliminary sample calculation tests based on available literature in order to minimize sample size [37]. Male, adult, New Zealand White rabbits were chosen, as young animals could bias the data by exhibiting increased potential for spontaneously repairing created bone defects and female animals could display hormonal interference [41].

Regarding the chosen intervention location, although skullcap healing model would embryologically be considered as the gold standard in bone graft material evaluation for dentistry, research shows a tendency for abnormal particle migration when different formulations are tested in skull models. For that reason, a model of critical defect, defined as the smallest bone defect that does not heal spontaneously, with less than 10% new bone formation during the life of the animal, in rabbit’s femoral condyle was chosen for this study [38,42,43,44]. The choice for this experimental model also granted the possibility to perform two defects per animal, thus reducing individual variability, economic costs and keeping animal numbers to a minimum [45].

Concerning the tested materials origin, Corbella et al. [46] published a systematic review and meta-analysis on histomorphometric results of postextraction socket healing with different biomaterials showing, among other conclusions, that when comparing to sites healed without bone substitutes, porcine-derived graft materials induced a significantly higher amount of new bone volume than bovine grafts. 

Considering the addition of collagen to bone substitutes, as presented by some of the tested materials, Nannmark et al. [28] reported an enhanced clinical handling and vital role in particle resorption mechanisms, as also stated by Mizuno et al. [28,47]. The specific influence of collagen on bone tissue behavior towards grafting material still needs more studies, but according to Barone et al. [48], it seemed to promote biomaterial resorption and operated a significant role in the material osteoconductive properties. Abdelgawad et al. [49] shed some light on possible collagen roles, showing that in a bone remodeling process, most newly recruited osteoblast lineage cells position directly beside osteoclasts exhibiting endocytic collagen receptors involved in collagen internalization and cell migration. The lack of these collagen receptors leads to an abrupt reduction of bone formation. Abdelgawad et al. [49] observations indicate that collagen demineralized by osteoclasts may play an haptotactic role in attracting osteoprogenitor cells to osteoclast consumed surfaces prolific in collagen receptors. This model may explain some of the results found in this research. Barone et al. [48] concluded by supporting the theory that collagenated porcine bone exhibits increased osteoconductive properties and is capable of being widely resorbed, as well as an increased percentage of newly formed bone and concomitant reduction in residual grafting material percentage. These results were corroborated by the present study. In terms of macrostructure and organization, a porous structure has shown to be the basis for any bone graft material [48]. Four of the five bone graft materials used in this experimental work have cortico-cancellous bone particles. However, the presentation of those particles varies, meaning that some have particle-only formulations, others exhibit particles mixed in various gel concentrations. These particles also diverge in size according to the studied material. Independently of the biomaterial, when performing bone regenerative procedures, not only particle characteristics matter but above all, their proper tissue distribution, compaction and location within the defect play a pivotal role. These features are directly related to particle size and shape, as well as the operator’s skill and experience in handling and accommodating the particles in the surgical defect, but also suffer major influence from the material itself and whether its presentation comprises a transporting medium or not. When excessively packed, granulated materials may act as blocks which will not allow their interstices to be invaded by cells. This concern underlies the idea of introducing a carrier vehicle that would predictably promote adequate particle spacing, as well as an easy usability by clinicians. Examples of these vehicles are the hydrogel formulations containing fewer mineral particles per volume and larger gaps in their matrix, as used in this study, theoretically being able to facilitate or even stimulate greater invasion of cellular and vascular components through larger interparticle spacing [50]. 

Comparing Apatos group with other tested materials, this cortical biomaterial presented the lowest amount of osteoclastic cells throughout the observed samples. When found, osteoclasts are adjacent to newly formed bone and very rarely around bone graft particles. Iezzi et al. [51]. described the same phenomena, which is in fact quite common in this type of bone regeneration materials, indicating a material low resorption rate. Barone et al. [52] compared a cortical porcine bone with a collagenated cortico-cancellous porcine bone (same as mp3 group) in several clinical trials with patients subjected to single-tooth alveolar ridge preservation. At the evaluation timepoint, the collagenated group presented a significantly lower reduction of ridge volume and a meaningfully smaller basal area shrinkage when compared to the noncollagenated group. The same author, in 2017 published two papers with randomized controlled trials corroborating the previous findings on the importance of these bone grafts [53,54]. Marconcini et al. [55] reached the same conclusion in their study about a 4-year randomized clinical trial of ridge-preserved versus nonpreserved sites. In the present study, histomorphometric results indicate that Apatos may perform comparably to other formulations, such as mp3 and Gen-Os, while presenting differences in terms of bone regeneration potential and particle presence when comparing to bone formulations like Putty and Gel 40.These results are also in accordance with Scarano et al. [56] and Orsini et al. [57].

At Gen-Os group, either at 15 or 30 days, all defect transversal sections displayed a remarkable and intense osteoclastic activity adjacent to significant amounts of osteoblast cells and osteoid matrix related to osteogenic phenomena, demineralizing, resorbing and disintegrating bone graft particles. Hence, it might be discussed that as a result, resorbed particle matrix may release various growth factors responsible for the proliferation and differentiation of pre-osteoblasts that migrate to this area from perivascular zones or bloodstream and create conditions for an effective colonization by cells of the osteoblastic line. Following these processes of osteoclastic degradation, several osteoinductive molecules (TGF, IGF-1, PDGF) contribute to the synthesis and mineralization of osteoid matrix [58,59,60]. In 2017, Iida et al. [61] published a histomorphometric experimental study in rabbits with a sinus-lifting technique using Gen-Os biomaterial and also described an intense resorptive processes substantiated by a high presence of multinucleated cells surrounding biomaterial particles which would be progressively resorbed at the 2 and 4 week periods. Iida et al. [62] published on this theme again in 2018 and reported intense osteoclastic activity when testing this biomaterial, resulting in a release of minerals and a possible increase in density of surrounding tissue. Addressing angiogenesis, Rombouts et al. [63] demonstrated a higher angiogenic potential when compared to Bio-Oss and suggests that Gen-Os may favor bone regeneration processes by stimulating early revascularization within grafted material. Histomorphometric analysis in the present study supports these observational findings, indicating that Gen-Os may perform comparably to other formulations, such as Apatos and mp3, presenting a reduction in particle percentage at 30 days, possibly due to resorption phenomena, but with no statistical significance. Scarano et al. [64] used a sheep iliac crestal bone defect and at 4-months healing period, defects where Gen-Os was used are described as completely filled by newly formed trabecular bone with bridging phenomena. Histomorphometry analysis showed newly formed bone mean percentages of 31.1 ± 1.9% and 23.4 ± 2.8% residual biomaterial particles. These findings are in accordance with the histomorphometry results of the present study. 

Concerning 90% porcine cortico-cancellous bone granulated mix with preserved collagen with 10% collagen gel (mp3), while performing the surgical part of this experimental study, the syringe presentation allowed for an easier handling of the material. This presentation appears to be an improvement over classical particulate materials and confers a safe and seemingly stress-free way of applying the material without wasting. The fact that this formulation is prehydrated also presents a major advantage for the clinician. Comparing mp3 group with other tested materials, in a strictly histological evaluation, this biomaterial seems to perform better than any other tested material at any timepoint. At 15 days the bridging phenomena with woven bone tissue even at the centre of the defect was evident and at 30 days a trabeculae network creates a continuity between opposite margins and also along the defect’s entire perimeter. Extensive areas of osteoblasts and osteoid in particle and trabeculae periphery reflect still ongoing osteogenic processes at both timeframes. Heterogeneous shapes, dimensions and tinctorial characteristics are also found in some granules, portraying particle resorption and demineralization process instigated by numerous osteoclasts broadly found adjacent to the biomaterial. These findings are in agreement with literature descriptions [26,48,65,66]. The results of histomorphometric analysis tend to corroborate what has been described in the preceding paragraphs. Noteworthy is the drastic variation in the percentage of particles between both timepoints, decreasing from 54.66 ± 3.51% to 25.96 ± 5.38%. This statistically significant variation (*p* = 0.009) may potentially be explained by the intense osteoclastic activity which leads to particle fragmentation and resorption. Regarding new bone formation, the mp3 group presented the highest percentages of all tested materials in both time periods with 40.93 ± 3.49% at 15 days and 52.49 ± 11.04% at 30 days. Barone et al. [48] reports newly formed bone percentages, using this biomaterial in sinus lifting procedures, of 30.7 ± 15.5% or 28.1 ± 19.4% depending on the surgical technique used. This same author, in a similar study design, shows areas of new formed bone of 43.95 ± 18.6% and 14.2 ± 13.6% of graft particles [65]. Guirado et al. [26] studied bone response to collagenated porcine xenografts in a tibia rabbit model and at 30 post-operatory days, newly formed bone represented 19.7 ± 1.5%. These findings seem to be short of what was found in the present experimental study. Another author, Giuliani et al. [66] described histological findings much similar to the ones found in this experimental study and histomorphometry showing an increase of bone and a decrease of residual biomaterial in the different timepoints. Nannmark et al. [28] used rabbit maxillary defects and also showed an intense osteoclastic activity surrounding mp3 particles with morphometric measurements displaying increased bone area with time, in parallel with a decrease of the graft area. The author’s explanation for these findings, alike ours, falls on the presence of osteoclasts resorbing the particles. In an attempt to compare mp3 with biomaterials of different origin, Silvestri et al. [67] compared this biomaterial to Bio-Oss. According to this author, both seem to present similar performances. 

Although the images from Putty and Gel 40 groups displayed in Figure 5, were chosen among the best results presented by both these materials, they seem prone to particle dispersion and migration with few to no bone formation beyond the peripheral zones of the defect at both 15 and 30 days of evolution, roughly corresponding to the natural regeneration expected in a critical dimension defect. At 30 days, Putty exhibited a mean of 12.58 ± 5.74% new bone formation and 1.02 ± 1.05% particle area. At the same timepoint, Gel 40 exhibited 11.65 ± 15.61% new bone formation and 0.66 ± 0.64% particle area. While applying the described methodology and performing the surgeries, Putty and Gel 40 syringe presentations and their doughy and plasticine-like handling properties allowed for effortless material handling. The only noticeable potential drawback observed was a complete stop on blood flow when Putty or Gel 40 were applied in the defect, which might facilitate in-surgery visualization, but may also be deemed to hinder the biomaterial colonization by blood, an essential step for bone regeneration success. Calvo-Guirado et al. [68] used Putty biomaterial graft in rabbit tibiae 4 mm diameter noncritical defects, analyzing data through radiography, histology and histomorphometry techniques at 1, 5, 8, and 15 months. Contrasting with our results, at 1 month these authors measured 20.7 ± 1.5% new bone and 28.8 ± 3.1% particles. At 5, 8, and 15 months bone percentage slightly raised, with a final mean of 27.32 ± 1.4% new bone. Nannmark et al. [28] used a study design with 5 × 8 × 3 mm bilateral bone defects created in the maxilla of female New Zealand White rabbits and filled them with Putty biomaterial. Histological and morphometrical evaluations were performed at 8 weeks and showed 42.3 ± 12.3% of new bone formation, being this the only result related to Putty biomaterial shown in the paper. Develioğlu et al. [69] used a model of cranial defect in rats to assess the short-term effects of Gen-Os and Gel 40 xenografts in bone healing. Although this study’s methodology implied a subjective histological evaluation supplemented by an histomorphometric analysis, little raw data is made available regarding this analytical methodology. Anyhow, a mean defect filling of 38.57 ± 6.9% was reported for Gel 40 and 27.5 ± 18.37% for Gen-Os. These findings presented no statistical significance and the authors concluded that both biomaterials used in the study are osteoconductive. The same authors published another paper in which a rat cranial defect model was also used, but the evaluation period was longer than in the aforesaid experiment, showing higher new bone formation in Gen-Os than in Gel 40 groups [70,71].

Regarding the discrepancies between our findings and some of the literature, we conjecture these differences may occur due to the demanding of our critical size defect or the different methodologies of experimental study design and evaluation, even though this experimental work attempted to mimic clinical conditions by using an heterologous pericardium membrane to cover the defect and prevent material displacement, which seems to have been effective in the Apatos, Gen-Os and mp3 groups. 

## 5. Conclusions

The tested porcine-derived bone graft materials presented favorable biocompatibility and appeared to undergo extensive resorption, demineralization and particle disintegration processes that may lead to biomaterial replacement by newly formed bone. All mp3, Gen-Os and Apatos exhibited promising results in terms of new bone formation, thus presenting suitable alternatives to be used in bone regeneration. Putty and Gel 40 show unfavorable histological outcomes when compared to the remaining biomaterials, with statistically significant histomorphometric differences when compared to mp3, regardless of the parameter and timepoint considered. 

## Figures and Tables

**Table 1:** Allocation of tested sites.

Material	Timepoint	
	15 Days	30 Days
Apatos	5 defects	5 defects
Gen-Os	5 defects	5 defects
mp3	5 defects	5 defects
Putty	5 defects	5 defects
Gel 40	5 defects	5 defects
Control	1 defect	1 defect
Total	26 defects	26 defects

**Table 2:** Group comparison regarding new bone formation percentage at both evaluated timepoints.

15 Days				
Material	Apatos	Gen-Os	mp3	Gel 40
Putty	0.505	0.106	0.016 *	1.000
Apatos		1.000	1.000	0.457
Gen-Os			1.000	0.093
mp3				0.014 *
**30 Days**				
**Material**	**Apatos**	**Gen-Os**	**mp3**	**Gel 40**
Putty	1.000	0.099	0.035*	1.000
Apatos		1.000	1.000	0.587
Gen-Os			1.000	0.046 *
mp3				0.015 *

* Statistically significant difference (*p* < 0.05); Dunn–Sidak post-hoc test with correction for multiple comparisons.

**Table 3:** Group comparison regarding particles percentage at both evaluated timepoints.

15 Days				
Material	Apatos	Gen-Os	mp3	Gel 40
Putty	0.240	0.135	0.003 *	1.000
Apatos		1.000	1.000	0.371
Gen-Os			1.000	0.215
mp3				0.006 *
**30 Days**				
**Material**	**Apatos**	**Gen-Os**	**mp3**	**Gel 40**
Putty	0.014 *	1.000	0.022 *	1.000
Apatos		0.932	1.000	0.007 *
Gen-Os			1.000	0.851
mp3				0.011 *

* Statistically significant difference (*p* < 0.05); Dunn–Sidak post-hoc test with correction for multiple comparisons.

## Data Availability

The data presented in this study are available on request from the corresponding author.

## Sample Availability

Samples of the compounds are not available from the authors.
